# Supplementary material for: Sources and Determinants of Wholegrain Intake in a Cohort of Australian Children Aged 12–14 Months
Source: Int J Environ Res Public Health. 2020 Dec 10;17(24):9229. doi: 10.3390/ijerph17249229 (PMC7764788; doi:10.3390/ijerph17249229)
Supplement: Supplementary file 1 [file ijerph-17-09229-s001.pdf]

International Journal of
Environmental Research and Public Health

Supplementary table S1. Sensitivity analysis: Factors independently[a] associated with usual wholegrain intakes (mean values and 95% confidence interval) of toddlers with plausible energy intakes ($n = 703$).

		Unadjusted mean (univariate)		Multivariable model
Maternal Characteristics		**g/day**	**95% CI**	***p***
Maternal age (years)	<25	13.8	11.0 – 16.7	0.001
	25-34	19.1	17.9 – 20.4	0.490
	≥35	18.8	16.9 – 20.6	REF
Maternal education	High school/vocational	18.6	17.1 – 20.1	0.412
	Some university and above	18.5	17.2 – 19.8	REF[3]
IRSAD[b]	Decile 1-2	18.1	15.4 – 20.7	0.609
	Decile 3-4	18.6	16.4 – 20.7	0.611
	Decile 5-6	19.9	17.4 – 22.5	0.747
	Decile 7-8	16.5	14.5 – 18.5	0.328
	Decile 9-10	19.6	17.5 – 21.7	REF
Maternal country of birth	Australia and New Zealand	20.0	18.9 – 21.1	REF
	UK	21.3	15.2 – 27.4	0.971
	India	17.9	14.2 – 21.7	0.285
	China	9.5	6.4 – 12.6	<0.001
	Asia-other[c]	12.1	8.9 – 15.4	<0.001
	Other[c]	15.1	11.8 – 18.5	0.026
Number of children	1	18.9	17.4 – 20.4	REF
	2	18.7	17.2 – 20.3	0.689
	≥ 3	17.5	15.0 – 19.9	0.063
Child Characteristics				
Sex	Male	19.3	17.9 – 20.7	0.071
	Female	17.6	16.3 – 19.0	REF
Age of introduction of complementary food (weeks)	< 17	18.8	16.6 – 20.9	0.949
	≥ 17	18.5	17.4 – 19.6	REF

[a] Multivariable linear regression adjusted for age of child at time of 24-h recall. [b] IRSAD, Index of Relative Socio-Economic Advantage and Disadvantage, where 1 = most disadvantaged and 10 = most advantaged. [c] No single country within this group had sample size sufficient for separate statistical analyses.
